# Impact of Ocular Complications on Visual Outcomes in Adult Patients With Uveitis

**DOI:** 10.7759/cureus.21370

**Published:** 2022-01-18

**Authors:** Maria del Mar Prieto del Cura, Juan Jacobo Gonzalez-Guijarro

**Affiliations:** 1 Ophthalmology, Hospital Infanta Leonor, Madrid, ESP; 2 Department of Ophthalmology, Hospital Universitario La Princesa, Madrid, ESP

**Keywords:** visual acuity, uveitis, retrospective cohort, complications, adult patients

## Abstract

Background

In this study, we aimed to assess the impact of ocular complications on visual outcomes in uveitis.

Methodology

We conducted a retrospective cohort study of 474 uveitis patients (655 eyes) with a mean age of 52.4 years who were followed for a median of 32 months (range: 8-80 months).

Results

At least one ocular complication was found in 317 eyes (48.4%), 161 of which were present at the time of diagnosis (prevalent complication). Although having an ocular complication was associated with a mean loss of 1.7 Early Treatment Diabetic Retinopathy Study (ETDRS) lines, the best-corrected visual acuity increased at the end of the study. Complications causing a decrease of ≥2 ETDRS lines were prevalent macular and peripheral retinal diseases, as well as new-onset corneal lesions, intraocular pressure alterations, and peripheral retinal diseases.

Conclusions

The impact of the most frequent complications (cataract and macular edema) did not reach two ETDRS lines. Macular diseases at presentation were the main risk factor for visual loss at the end of follow-up.

## Introduction

Uveitis is a term used to describe a group of intraocular inflammatory diseases that can occur at any age but predominantly affect patients in the working-age group. The average annual incidence of uveitis has been reported to be approximately 14-17 per 100,000, rising to a peak in the 20-50-year age group, following which it declines [1,2].

Uveitis can cause devastating visual loss and is the fifth most common cause of visual loss in the developed world, accounting for up to 20% of legal blindness in the Western world and 25% in developing countries [3-5]. The World Health Organization (WHO) has defined blindness as the best-corrected vision in the better eye of less than 3/60 or a visual field of ≤10°. On the other hand, severe visual impairment is defined as the best-corrected visual acuity (BCVA) in the better eye of 3/60 or more, but less than 6/60 or a visual field ≤20°. Legal blindness can be defined in some countries as the level of blindness that makes a person eligible for social support and financial benefits. Blindness caused by uveitis is potentially treatable. Furthermore, with a majority of patients in the working-age group, the impact on the quality of life and the potential social and economic costs are tremendous [6,7].

Uveitis is classified by the primary anatomic location of the inflammation according to the Standardization of Uveitis Nomenclature (SUN) guidelines, as well as whether it is caused by an infectious agent or is associated with an immune-mediated disease [8]. Noninfectious intermediate uveitis, posterior uveitis, or panuveitis are less common than anterior uveitis and are associated with a substantial risk of ocular complications, particularly in patients with persistent disease [9,10].

The main sight-threatening complications of uveitis include cataract formation, glaucoma, and maculopathy. These complications show an increasing prevalence in the normal elderly population.

Few studies have documented the frequency of visual loss in the general uveitis population [10,11]. In a 1962 hospital-based study from Minnesota, Darrell et al. [11] found that the rate of visual loss in patients with uveitis was 6%. Rothova et al. [10] found a similar rate of visual loss in a mixed primary and secondary referral center over 30 years later. The latter study found cystoid macular edema, corneal opacities, and macular inflammatory lesions to be the major causes of visual loss resulting from intraocular inflammation. In a primary referral cohort of 561 consecutive uveitis patients attending three district hospitals in the United Kingdom, the visual loss was associated with age at onset of >60 years, long follow-up, and a history of cataract surgery, with visual impairment being less likely in patients with acute anterior uveitis [12]. Among participants of the Systemic Immunosuppressive Therapy for Eye Diseases cohort study, posterior synechiae, active uveitis, and prior intraocular surgery were statistically significantly associated with decreased visual acuity (VA), both at presentation and during follow-up, whereas the use of immunosuppressive drugs was associated with a reduced risk of visual loss [13].

However, in none of the previous studies, a distinction has been made between the impact of prevalent ocular complications (those already present at diagnosis of uveitis) and incident complications (new-onset events at follow-up) on visual outcome. Therefore, this study aimed to assess the effect of prevalent and incident ocular complications on VA in a large series of adult patients with uveitis.

## Materials and methods

Study design and participants

In this retrospective longitudinal cohort study, the clinical records of 474 consecutive newly referred uveitis patients (655 eyes) who were examined at the Department of Ophthalmology at La Princesa University Hospital, Madrid (Spain) between May 1, 1989, and December 31, 2018, were reviewed. The primary objective of the study was to assess the impact of ocular complications present at the time of diagnosis or referral (prevalent complications) or developed at follow-up (incident complications) on the visual outcome. The secondary objective was to determine whether demographic and clinical factors affect VA. Data were extracted from patients’ medical records and prospectively entered into a database (FileMaker Pro, FileMaker Inc., Santa Clara, CA, USA). The study protocol was approved by the Institutional Review Board, and the research followed the tenets of the Declaration of Helsinki principles.

Eligible participants were identified according to the following inclusion criteria: 18 years of age or older, presence of active uveitis, referred for diagnostic investigations and/or treatment, and follow-up in our center for at least three months after referral or diagnosis. Patients with scleritis and those with visual loss due to causes other than uveitis were excluded from the study.

Study procedure and data collection

All patients underwent a complete history, comprehensive ocular examination, and protocolized diagnostic workup to determine the cause of uveitis. The characteristics of uveitis were analyzed according to the definitions of the SUN criteria [8-14]. Before the publication of the SUN criteria in 2005, the chart findings were translated into SUN terms for analysis.

For each patient, the following data were recorded: demographic characteristics, ophthalmological variables, etiology of uveitis, treatment, and complications. The following ophthalmological variables were included: (a) anatomic location of inflammation including anterior uveitis (iritis, iridocyclitis, and anterior cyclitis), intermediate uveitis (para planitis, posterior cyclitis, and hyalitis), posterior uveitis, and panuveitis; (b) clinical course described as acute, recurrent, chronic, or indeterminate; (c) laterality (unilateral, bilateral); and (d) etiology of uveitis (infectious, ocular entities, systemic diseases, postsurgical, posttraumatic, drug-induced, and undetermined). The degree of activity of inflammation of the anterior chamber cells and the vitreous was graded according to the SUN criteria [8-14] and the National Eye Institute [15], respectively. Active uveitis was defined as grades 0.5+ and higher of inflammation of the anterior chamber cells and the vitreous cavity. The duration of inflammation recorded in the last visit was categorized as <three months or ≥three months (persistent uveitis) [8-14].

Complications were classified into cataract, cystoid macular edema (CME); macular diseases (choroidal neovascular and epiretinal membranes, macular atrophy, macular hole, macular ischemia, and macular scar); diseases of the peripheral retina (retinal tears, rhegmatogenous, and/or tractional retinal detachment); retinal vascular diseases (retinal or retinovitreous neovessels, retinal vascular occlusions); corneal alterations (endothelial decompensation, corneal opacity, band keratopathy); iris alterations (minor or major [>180°] posterior synechiae, atrophy of the iris sphincter or stroma, block and occlusion of the pupil); intraocular pressure (IOP) alterations (elevated at >24 mmHg, hypotony <7 mmHg) measured using Goldmann applanation tonometry. Glaucoma was diagnosed when typical visual field defects or optic neuropathy were observed. The diagnosis of CME was made using the Goldmann three-mirror lens examination and was confirmed by fluorescein angiography. Optical coherence tomography (OCT) using Stratus OCT (Carl Zeiss Meditec Inc., Dublin, CA, USA) and Spectralis OCT (Heidelberg Engineering, Vista, CA, USA) since 2004 and 2011, respectively, was used to determine the presence of CME. A similar diagnostic approach was used for macular and retinal diseases.

BCVA expressed as the logarithm of the minimal angle of resolution (logMAR) units was obtained at the first visit and the final VA was recorded in patients’ medical history. BCVA was assessed using the same Early Treatment Diabetic Retinopathy Study (ETDRS) optotype chart illuminator cabinet, with ETDRS charts from Precision Vision (La Salle, IL, USA) since 2004 (prior to its acquisition, VA was measured in a decimal scale using Takagi ophthalmic chart projector [Takagi Seiko Co., Ltd., Nagano, Japan] and converted to logMAR using the formula [16]: decimal acuity = antilog (-logMAR) = 10-logMAR). For patients with very low vision, the following logMAR values were established: “counting fingers” = 2.0 logMAR, “hand motion” = 2.3 logMAR, “light perception” = 2.6 logMAR, and “no light perception” = 2.9 logMAR [17]. The visual outcome was assessed according to the difference between the final and initial BCVA and arbitrarily categorized as 0 = no change, 1 = a gain of ≥2 ETDRS lines, 2 = a gain of < 2 ETDRS lines, 3 = a loss of <2 ETDRS lines, and 4 = a loss of ≥2 or more ETDRS lines. Visual impairment was also categorized using the SUN criteria [8,14] as follows: normal ≥1 decimal, 20/20 Snellen, 0 logMAR; mild >0.4-0.9 decimal, 20/50-20/22 Snellen, <0.4-0.05 logMAR; moderate 0.4-0.1 decimal, 20/50-20/200 Snellen, 0.4 to <1 logMAR; and severe ≤0.10 decimal, ≤20/200 Snellen, ≥1 logMAR.

Statistical analysis

Categorical variables were expressed as frequencies and percentages and quantitative variables as mean and standard deviation (SD) or median and interquartile range (IQR) (25-75th percentile). The absolute effect of ocular complications (mean final - initial BCVA), the mean visual loss associated with each complication, independent variables associated with the final BCVA, and those associated with a loss of two or more ETDRS lines were calculated. The Student’s t-test for paired data was used to assess the mean absolute effects of complications, and covariance models were used to determine variables related to the final BCVA adjusted by the initial BCVA. Linear regression models adjusted by the initial BCVA and the length of follow-up were used to assess the changes in BCVA according to the presence or absence of complications. Variables independently associated with the mean final BCVA were assessed using a logistic regression model adjusted by the initial BCVA, age, and variability of the two patient’s eyes. Risk factors for a visual loss of ≥2 ETDRS lines were determined using a conditional logistic regression model adjusted by the initial BCVA and age. Odds ratio (OR) and 95% confidence intervals (CIs) were calculated to assess the probability of the final BCVA with a loss of two or more ETDRS lines (category 4). Variables independently associated with the categories of visual outcome were analyzed in a logistic regression model adjusted by the initial BCVA and length of follow-up. Data were analyzed using the Statistical Package for the Social Sciences (SPSS) version 20.0 (IBM Corp., Armonk, NY, USA). Statistical significance was set at P-values of <0.05.

## Results

Patient characteristics

There were 216 men and 258 women (54.4%), with a mean (SD) age of 51.0 (19.1) years. The clinical characteristics of patients are shown in Table 1.

**Table 1:** Clinical characteristics of 474 patients with uveitis. Data expressed as frequencies and percentages in parenthesis unless otherwise stated. SD: standard deviation; HLA-B27: human leukocyte antigen B27

Variables	Number of patients (%)
Gender	
Men	216 (45.6)
Women	258 (54.4)
Age, years, mean (SD)	51.0 (19.1)
Toxic habits, n = 407	144 (35.4)
Smoking habit	108 (75.0)
Alcohol consumption	9 (6.2)
Previous diseases, n = 474	
Systemic disorders	238 (50.2)
Autoimmune diseases	101 (42.4)
Cardiovascular diseases	97 (40.8)
Tumors	22 (9.2)
Other	18 (7.6)
Infectious diseases	140 (29.5)
Viruses	60 (42.8)
Bacteria	35 (25.0)
Other	21 (15.0)
Anatomic localization	
Anterior uveitis	234 (49.4)
Intermediate uveitis	59 (12.4)
Posterior uveitis	122 (25.7)
Panuveitis	59 (12.4)
Bilateral disease	216 (33.1)
Clinical course, n = 457	
Acute	169 (37.0)
Chronic	154 (33.7)
Recurrent	134 (29.3)
Indeterminate	28 (5.9)
Etiology	
Associated with systemic diseases	108 (22.8)
Ankylosing spondylitis HLA-B27 negative	28 (5.9)
HLA-B27-associated uveitis	17(3.6)
Sarcoidosis	11 (2.3)
Behcet’s disease	10 (2.1)
Multiple sclerosis	7 (1.5)
Rheumatoid arthritis	6 (1.3)
Seronegative spondyloarthropathy	7 (1.5)
Masquerade syndrome	3 (0.6)
Juvenile idiopathic arthritis	4 (0.8)
Inflammatory bowel disease	4 (0.8)
Vogt-Koyanagi-Harada syndrome	3 (0.6)
Psoriasis	1 (0.2)
Sjögren’s syndrome	2 (0.4)
Diabetes mellitus	2 (0.4)
Seronegative arthritis	1 (0.2)
Reiter syndrome	1 (0.2)
Polychondritis	1 (0.2)
Etiology (continued)	7 (1.5)
Established ocular entity	59 (12.4)
Fuchs’ heterochromic cyclitis	17 (3.6)
Pars planitis	12 (2.5)
Glaumatocyclitic crisis	8 (1.7)
Acute posterior multifocal placoid pigment epitheliopathy	6 (1.3)
Eales’ disease	4 (0.8)
Evanescent white dot syndrome	5 (1.1)
Punctate inner choroidopathy	5 (1.1)
Birdshot chorioretinopathy	6 (1.3)
Phacogenic uveitis	3 (0.6)
Sympathetic ophthalmia	1 (0.2)
Serpiginous choroiditis	1 (0.2)
Recurrent branch retinal artery occlusions	1 (0.2)
Infectious	163 (34.4)
Viruses	75 (15.8)
Protozoa/parasites	40 (8.4)
Bacteria	45 (9.5)
Fungi	3 (0.6)
Postsurgical	21 (4.4)
Drug-induced	4 (0.8)
Post-traumatic	1 (0.2)
Undetermined	108 (25.8)
Treatment, n = 655 eyes	
Topical steroids	191 (29.2)
Posterior subtenon steroid injections	152 (23.4)
Dexamethasone intravitreal implant	11 (1.3)
Intravitreal injections	60 (9.2)
Oral corticosteroids	290 (44.8)
Oral non-steroidal anti-inflammatory drugs	115 (17.8)
Anti-infectious agents	204 (31.4)
Disease-modifying antirheumatic drugs (DMARDs)	113 (17.5)
Ocular surgery	139 (21.2)

Anterior uveitis was the most frequent (49.4%), followed by posterior uveitis (25.7%). In 33.1% of the cases, uveitis was bilateral. Acute uveitis was present in 37.0% of the patients, chronic uveitis in 33.7%, and recurrent uveitis in 29.3%. Infectious uveitis was diagnosed in 34.4% of the patients, uveitis associated with systemic diseases in 22.8%, uveitis due to an established ocular entity in 12.4%, and undetermined uveitis in 25.8%. Treatment included oral corticosteroids in 44.8%, topical steroids in 29.2% of the eyes, posterior sub-Tenon’s steroid injections in 23.4%, intravitreal injections in 9.2%, and dexamethasone intravitreal implant in 1.3%. Disease-modifying anti-rheumatic drugs (DMARDs) were administered to 17.5% of the patients. Ocular surgery was recorded in 21.2% of the cases.

Total complications and visual outcome

**Figure 1:**
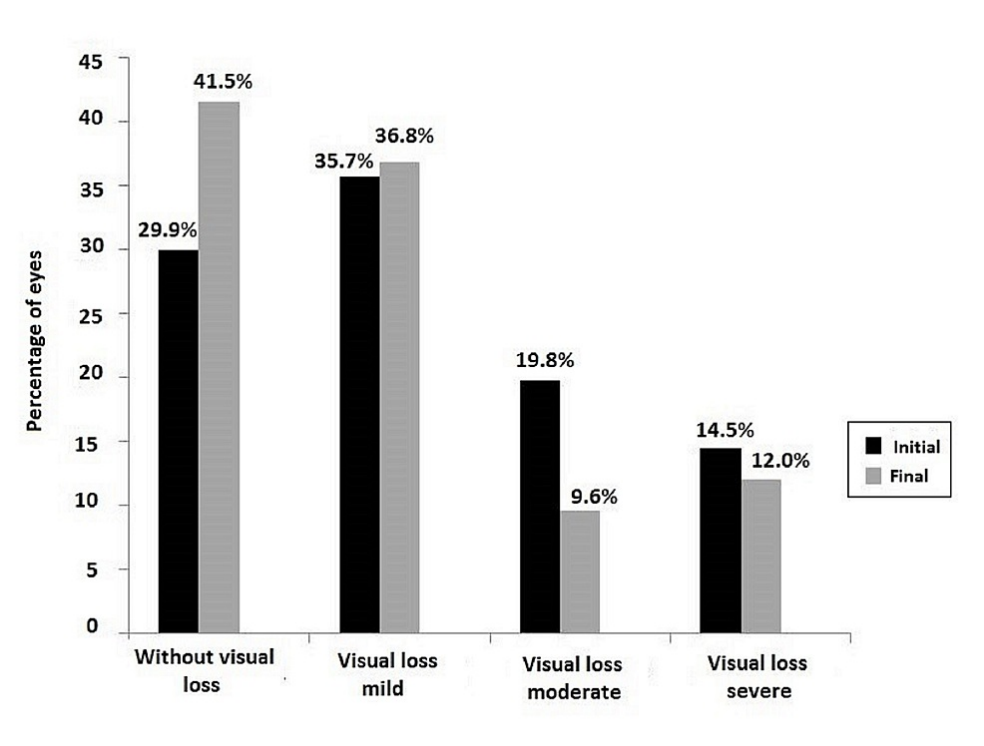
Classification of the initial and final BCVA according to the SUN criteria. BCVA: best-corrected visual acuity; SUN: Standardization of Uveitis Nomenclature

The median follow-up of the entire cohort was 32 months (IQR: 8-80 months). The initial mean BCVA was 0.36 (0.45) logMAR and the final mean BCVA was 0.27 (0.42) logMAR, with a gain of almost one ETDRS line (0.9 logMAR, 95% CI: 0.6-1.2 logMAR; P < 0.001). According to the SUN categories, there was a decrease of eyes with moderate and severe visual impairment at the end of the study and an increase of eyes with normal vision or mild visual impairment (Figure 1).

There were 317 eyes with ocular complications for a total of 489 complications.The mean gain of BCVA in eyes without complications was one ETDRS line (95% CI: 0.8-1.4; P < 0.001) compared with a gain of 0.7 ETDRS lines (95% CI: 0.2-1.2, P = 0.004) in eyes with complications. The distribution of ocular complications and BCVA for each type of complication are shown in Table 2.

**Table 2:** Distribution of 489 ocular complications in the total cohort of 655 eyes and changes in BVCA. One patient with acute retinal necrosis presented bilateral blindness (visual acuity ≤ 20/400) at the end of the study. SD: standard deviation; CI: confidence interval; IOP: intraocular pressure; BCVA: best-corrected visual acuity; logMAR: logarithm of the minimal angle of resolution

Complication	Number	Initial BCVA, logMAR mean (SD)	Final BCVA, logMAR mean (SD)	Absolute effect, logMAR mean (95% CI)	P-value
Cataract	130	0.53 (0.58)	0.48 (0.50)	0.05 (-0.04 to 0.14)	0.294
Iris alterations	97	0.45 (0.46)	0.37 (0.47)	0.07 (-0.02 to 0.17)	0.117
Macular edema	81	0.53 (0.40)	0.40 (0.42)	0.13 (0.02 to 0.23)	0.020
Macular diseases	59	0.74 (0.56)	0.68 (0.54)	0.06 (-0.06 to 0.19)	0.322
IOP alterations	51	0.54 (0.53)	0.52 (0.54)	0.02 (-0.10 to 0.14)	0.701
Peripheral retinal diseases	36	0.61 (0.53)	0.68 (0.59)	-0.07 (-0.24 to 0.10)	0.417
Retinal vascular diseases	28	0.46 (0.46)	0.38 (0.51)	0.08 (-0.11 to 0.27)	0.416
Corneal alterations	15	0.75 (0.60)	0.75 (0.54)	-0.01 (-0.35 to 0.34)	0.961

**Figure 2:**
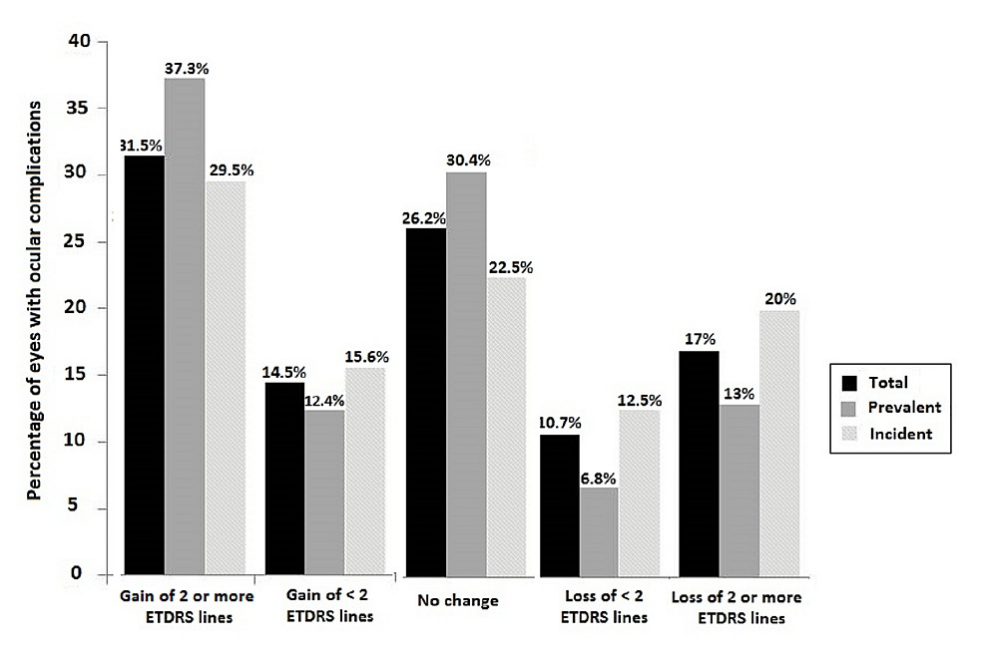
Changes of the final BCVA compared to the initial BCVA in eyes with ocular complications. BCVA: best-corrected visual acuity; ETDRS: Early Treatment Diabetic Retinopathy Study

Differences between the final and initial mean BCVA (absolute effect) were not statistically significant, except for macular edema in which a gain of 1.3 ETDRS lines at the end of the study was observed (P = 0.020). As shown in Figure 2, BCVA was maintained or improved in 72.2% of the eyes with ocular complications, with gains of ≥2 ETDRS lines in 31.5% of the cases and <2 ETDRS lines in 14.5%. Loss of ≥2 ETDRS lines was recorded in 17% of the cases.

Prevalent complications and visual outcome

There were 161 eyes with prevalent ocular complications for a total of 202 prevalent complications (Table 3).

**Table 3:** Ocular complications in 489 eyes from 474 patients with uveitis. Percentages in parenthesis.

Complications	Prevalent (n = 202)	Incident (n = 287)
Cataract	42 (20.8)	88 (30.7)
Iris alterations	61 (30.2)	40 (13.9)
Posterior synechiae <180°	27 (13.4)	21 (7.3)
Posterior synechiae ≥180°	31 (15.3)	14 (4.9)
Pupillary block	2 (1.0)	5 (1.7)
Iris sphincter atrophy	1 (0.5)	0
Macular edema	30 (14.8)	51 (17.8)
Macular diseases	13 (6.4)	48 (16.7)
Neovascular membrane	6 (3.0)	7 (2.4)
Epiretinal membrane	5 (2.5)	31 (10.8)
Macular atrophy	0	5 (1.7)
Macular necrosis	2 (1.0)	5 (1.7)
Intraocular pressure alterations	22 (10.9)	29 (10.1)
Ocular hypertension	17 (8.4)	14 (4.9)
Glaucoma	5 (2.5)	12 (4.2)
Ocular hypotony	0	3 (1.0)
Peripheral retina and vitreous alterations	11 (5.4)	26 (9.0)
Retinal detachment	6 (3.0)	16 (5.6)
Vitreous opacity	4 (2.0)	3 (1.0)
Retinal tears	1 (0.5)	7 (2.4)
Retinal vascular diseases	16 (7.9)	12 (4.2)
Venous retinal occlusion	9 (4.5)	5 (1.7)
Vitreous hemorrhage	4 (2.0)	4 (1.4)
Retinal neovessels	3 (1.5)	3 (1.0)
Corneal alterations	7 (3.5)	8 (2.8)
Band keratopathy	5 (2.5)	4 (1.4)
Corneal opacity	1 (0.5)	0
Corneal edema	1 (0.5)	0
Corneal decompensation	0	4 (1.4)

The median follow-up of patients with prevalent complications was 25 months (IQR: 8-64 months). The initial BCVA was 0.58 (0.48) logMAR and the final BCVA was 0.47 (0.52) logMAR, with a gain of 1.1 ETDRS lines (95% CI: 0.4-1.7 logMAR; P = 0.001).

Table 4 shows the distribution of prevalent complications and BCVA changes for each individual complication. Differences between the final and initial BCVA were not statistically significant, except for macular edema with a mean gain of 1.8 ETDRS lines (P = 0.026). At the end of the study, BCVA was maintained or improved in 80% of the eyes, with a gain of ≥2 ETDRS lines in 37.3% of cases and <2 ETDRS lines in 12.4% (Figure 2). Loss of ≥2 ETDRS lines was recorded in 13% of the cases and of <2 ETDRS lines in 6.8%.

**Table 4:** Distribution of 202 prevalent ocular complications (161 eyes) and 287 incident ocular complications (200 eyes) and changes in BVCA. SD: standard deviation; IOP: intraocular pressure; BCVA: best-corrected visual acuity; logMAR: logarithm of the minimal angle of resolution

Complication	Number	Initial BCVA, logMAR mean (SD)	Final BCVA, logMAR mean (SD)	P-value
Prevalent complications				
Iris alterations	59	0.51 (0.49)	0.39 (0.4)	0.058
Cataract	42	0.71 (0.49)	0.56 (0.52)	0.105
Macular edema	30	0.54 (0.29)	0.36 (0.40)	0.026
IOP alterations	22	0.53 (0.53)	0.36 (0.49)	0.077
Retinal vascular diseases	15	0.39 (0.43)	0.37 (0.55)	0.733
Macular diseases	12	0.91 (0.42)	1.06 (0.45)	0.238
Peripheral retinal diseases	11	0.62 (0.60)	0.72 (0.74)	0.555
Corneal alterations	7	0.64 (0.53)	0.53 (0.43)	0.484
Incident complications				
Cataract	88	0.44 (0.45)	0.44 (0.49)	0.941
Macular edema	51	0.52 (0.46)	0.42 (0.44)	0.188
Macular diseases	47	0.70 (0.58)	0.58 (0.52)	0.111
Iris alterations	38	0.34 (0.38)	0.33 (0.47)	0.856
IOP alterations	29	0.56 (0.53)	0.64 (0.54)	0.247
Peripheral retinal diseases	25	0.60 (0.51)	0.66 (0.54)	0.583
Retinal vascular diseases	13	0.53 (0.49)	0.44 (0.52)	0.630
Corneal alterations	8	0.84 (0.68)	0.95 (0.58)	0.696

The linear regression model showed that the presence of prevalent complications caused a mean visual loss of 1.2 ETDRS lines (95% CI: 0.6-1.8) compared to the absence of prevalent complications (P < 0.001). According to the type of complications, significant differences for the presence or absence were found for macular diseases, peripheral retinal diseases, and cataracts (Table 5).

**Table 5:** Results of linear regression analysis: visual loss according to the type of prevalent and incident ocular complications. CI: confidence interval; IOP: intraocular pressure; ETDRS: Early Treatment Diabetic Retinopathy Study

Ocular complications	Loss of ETDRS lines (95% CI)	P-value
Prevalent		
Macular diseases	5.1 (3.3 to 7.0)	<0.001
Peripheral retinal diseases	3.3 (1.3 to 5.2)	0.001
Cataract	1.3 (0.3 to 3.3)	0.014
Incident		
Corneal alterations	4.1 (1.8 to 6.5)	<0.001
IOP alterations	2.7 (1.5 to 4.0)	<0.001
Peripheral retinal diseases	2.7 (1.4 to 4.0)	<0.001
Cataract	1.6 (0.8 to 2.3)	<0.001
Macular diseases	1.5 (0.5 to 2.5)	0.003

Incident complications and visual outcome

There were 200 eyes with incident ocular complications for a total of 287 incident complications (Table 2). The median follow-up of patients with incident complications was 58 months (IQR: 32-96 months). The initial BCVA was 0.48 (0.49) logMAR and the final BCVA was 0.42 (0.49) logMAR, with a gain of 0.6 ETDRS lines. Differences between the final and initial BCVA were not statistically significant for any type of incident complication (Table 4). At the end of the study, BCVA was maintained or improved in 68% of the eyes with incident complications (gain of ≥2 ETDRS lines in 29.5%, gain of <2 ETDRS lines in 15.6%). Loss of ≥2 ETDRS lines was recorded in 20% of eyes and <2 ETDRS lines in 12.5% (Figure 2).

The linear regression model showed that the presence of incident complications caused a mean visual loss of 1.4 ETDRS lines (95% CI: 0.8-2.0) compared to the absence of complications (P < 0.001). According to the type of complications, significant differences for the presence or absence were found for corneal alterations, alterations of IOP, cataracts, macular diseases, and peripheral retinal diseases (Table 5).

Variables associated with final BCVA and visual loss of ≥2 ETDRS lines

As shown in Table 6, in the multivariate analysis, variables independently associated with the final BCVA were prevalent and incident macular diseases, incident IOP alterations, incident cataract, intravitreal therapy, treatment with oral corticosteroids, and viral/fungal etiology of uveitis. Moreover, risk factors for a final visual loss of ≥2 ETDRS lines were prevalent and incident macular diseases, incident cataract, incident macular edema, intravitreal therapy, treatment with oral corticosteroids, and viral/fungal etiology of uveitis.

**Table 6:** Multivariate analysis: variables associated with the final BCVA and risk factors for visual loss of ≥2 ETDRS lines. CI: confidence interval; IOP: intraocular pressure; BCVA: best-corrected visual acuity; ETDRS: Early Treatment Diabetic Retinopathy Study

Variables	Final BCVA		Loss of ≥2 ETDRS lines	
	Loss of ETDRS lines (95% CI)	P-value	Odds ratio (95% CI)	P-value
Prevalent macular diseases	6.4 (8.4 to 4.4)	<0.001	12.96 (2.85 to 58.96)	0.001
Incident macular diseases	1.2 (2.1 to 0.2)	0.019	2.45 (1.02 to 5.89)	0.046
Incident cataract	1.3 (2.0 to 0.5)	0.001	2.71 (1.39 to 5.28)	0.003
Intravitreal therapy	1.2 (2.2 to 0.2)	0.015	6.0 (2.67 to 13.69)	<0.001
Oral corticosteroids	0.6 (11 to 0)	0.32	2.26 (1.25 to 4.06)	0.007
Viral/fungal etiology of uveitis	0.2 (0.4 to 0)	0.024	1.39 (1.18 to 1.64)	<0.001
Incident IOP alterations	1.9 (3.2 to 0.7)	0.002		

## Discussion

This retrospective cohort study analyzed the impact of ocular complications on visual outcome in a clinical series of 655 eyes of 474 adult patients with uveitis. After a median follow-up of 32 months, having a complication was associated with a mean loss of 1.7 ETDRS lines. However, there was an increase in BCVA at the end of the study, which was independent of the presence or absence of complications and can be attributed to the effect of treatment. The mean initial BCVA was 0.36 (0.45) logMAR and the final BCVA was 0.27 (0.42) logMAR. This overall favorable visual outcome is consistent with results reported in other studies [18-20]. In a cross-sectional study of 305 patients (550 eyes) selected from a database of uveitis patients, median BCVA was 0.26 (0.38) at presentation, 0.22 (0.42) at five years, and 0.23 (0.46) at 10 years [19]. In a retrospective observational study of 491 uveitis patients (644 eyes) seen at the University of Virginia from 1984 to 2014, the median overall BCVA was logMAR 0.18 at the initial and final presentations after a mean duration of follow-up of 4.8 years [18]. In a cross-sectional study of 1,076 patients (1,799 eyes) who attended the uveitis clinic at Moorfields Eye Hospital in London between 2011 and 2013, average BCVA remained stable for patients with both anterior and non-anterior uveitis after a median follow-up of 5.6 years [20]. In a cohort of 133 newly referred patients with active uveitis to a tertiary center, the majority of patients developed ocular complications and (temporary) decreased vision during the first year, with a substantial number of patients requiring systemic treatment and intraocular surgery [21]. However, the visual results at the end of the first year were favorable, with only 4% of patients having bilateral visual impairment, probably as a reflection of the intensive treatment of their patients.

In our study, in addition to various local treatment modalities (including sub-Tenon’s steroid injections, dexamethasone implants, and intravitreal injections), 45% of patients received systemic corticosteroids and 17% either non-steroidal anti-inflammatory drugs or DMARDs. However, a comparison of treatment modalities and their effects on visual outcome was not performed. We found an association between the use of oral steroids and a decrease of 0.6 ETDRS lines as well as a 2.26-fold increase in the risk of loss of ≥2 ETDRS lines. Tomkins-Netzer et al. reported a relationship between the use of oral steroids and moderate or severe visual loss. Intravitreal treatment was associated with visual loss of 1.2 ETDRS lines and a six-fold increase in the risk of loss of ≥2 ETDRS lines. Similar associations have not been reported in the literature either in the case of oral corticosteroid or intravitreal therapy, and these results may be explained as a possible consequence of the severity of uveitis and/or the presence of complications [20].

An interesting design of this study is the assessment of ocular complications categorized as prevalent and incident (new-onset) and the differential impact on BCVA. This aspect has not been considered in previous studies. It is possible that the greater impact of prevalent complications may occur in patients with more advanced and/or severe uveitis, with late diagnosis or inadequate treatment. In a retrospective cohort study of 96 patients (174 eyes) with intermediate uveitis, with a mean follow-up of 64.9 months, most sight-threatening complications (namely, CME and glaucoma) were diagnosed at presentation, suggesting that despite the low-grade nature of the inflammation, it was of sufficient magnitude and duration to induce the development of ocular complications in an insidious manner [22].

In our study, macular diseases were the main cause of visual loss, particularly prevalent maculopathies (5.1 ETDRS lines), with a lower impact of incident macular diseases (loss of 1.5 ETDRS). This type of complication has already been described as one of the main causes of moderate visual loss, including macular scar formation or atrophy secondary to retinochoroidal scar of non-infectious uveitis, choroidal neovascularization, or chronic macular edema [23,24]. Visual impairment is associated with the duration and severity of macular edema [12]. In the retrospective cohort study of 133 newly referred uveitis patients with active uveitis reported by Groen et al., macular edema and epiretinal membrane were the most common new complications, which may be related to the early detection of these complications by the introduction of the OCT scanning technique [21]. In our series of patients, it may be possible that mild cases of different macular complications could not have been diagnosed prior to the use of OCT in routine practice.

Incident corneal complications in our series were associated with a mean loss of 4.1 ETDRS lines. In the series of 582 patients with uveitis who visited the ophthalmology departments of two university hospitals in the Netherlands in 1993, the most important cause of visual loss was CME followed by corneal opacities [10]. However, corneal alterations are uncommon complications that arise in long-standing uveitis but are rarely analyzed as a cause of vision loss in general series of patients with uveitis. It has been shown that acute anterior uveitis causes morphological corneal endothelium changes, such as endothelium cells density loss, increased pleomorphism, and polymegathism, thus compromising endothelial and visual function [25,26].

Prevalent and incident peripheral retinal diseases, especially rhegmatogenous retinal detachment (RRD), were the third cause of loss of ETDRS lines, the impact of which does not seem to be different in relation to the time of diagnosis (prevalent or incident). Retinal detachment is a common and severe complication among patients with uveitis, with a prevalence of RRD of approximately 3%, more frequently associated with posterior uveitis and infection causes, particularly viral infections [27]. The visual outcomes among patients with RRD and uveitis are worse compared to outcomes in patients with non-uveitic RRD. Tomkins-Netzer et al. described severe vision loss (≤20/200) due to RRD in 1.3% of 1,076 uveitis patients [20].

Incident IOP alterations, including glaucoma, caused a loss of almost three ETDRS lines. In the study by Bajwa et al. including 644 eyes of 491 patients, ocular hypertension was associated with moderate and severe visual loss, whereas hypotony was not [18]. In this study, when increased IOP was present at baseline, the odds that the eye had moderate or severe visual loss was 1.89 times than the odds for an eye that was normotensive, which increased to 2.62 times when the eye was hypertensive at follow-up. The incidence of uveitic glaucoma is reported to be 10-20%, and it is difficult to manage inflammation and elevated IOP simultaneously [28].

Cataract was the most common ocular complication but had a limited impact on visual outcome. However, the visual morbidity of this complication should not be underestimated. Our results could have been influenced by the design of the study in which final BCVA values were recorded before cataract surgery. However, in the study by Durrani et al., cataract and CME either alone or in combination were responsible for visual loss in 64.5% of the patients [5]. Rothova et al. investigated the causes of visual loss in 582 patients with intraocular inflammation and found CME to be the most common cause of decreased vision (26%) followed by cataracts (19%) [10].

When the absolute effect of complications was analyzed, macular edema was the only complication with significant differences between the final and initial BCVA, with visual gains at the end of the study of 1.8 ETDRS lines for prevalent macular edema and one ETDRS line for incident macular edema. This is probably related to the efficacy of vigorous treatment. Macular edema is one of the most common complications in uveitis, especially in intermediate, posterior, or panuveitis. In a cross-sectional study of 529 patients (842 eyes) with uveitis reported by Lardenoye et al., CME was a major complication causing visual loss in uveitis, being particularly severe among elderly patients and those with chronic disease [29]. In our study, among the eyes with significant visual loss, macular edema was the second more frequent complication after cataract. Routine use of OCT, early diagnosis, and aggressive treatment have improved the prognosis of visual outcomes associated with this complication.

In our study, the viral etiology of infectious uveitis was a predictive factor for decreased BCVA and showed a 1.39-fold increased risk of loss of ≥2 ETDRS lines. To our knowledge, this association has not been previously recorded in the literature. Of note, there was a high percentage of infectious uveitis (34%) in our series.

Limitations of the study include those common to retrospective studies conducted over a prolonged period of time. It is possible that there have been changes in the diagnostic approach, workup studies, and treatment strategies in patients with uveitis, although the single-center nature of the study reduced the variability associated with multicenter designs. Additionally, because the study was retrospective, the findings should be interpreted as ocular complications that seem to be related with a greater risk for visual loss in our cohort rather than a causal association. However, an important contribution of the study is the use of ETDRS lines as a measure of visual loss, which makes the comparison of these quantitative changes with those of other series challenging, in which visual gains or loss in ETDRS were not reported.

## Conclusions

In the present series of patients with uveitis, the impact of the most frequent complications (cataract and macular edema) did not reach two ETDRS lines. Prevalent macular diseases detected at presentation were the main risk factor for visual loss at the end of follow-up. A final mean BCVA greater than 0.4 decimal in almost 80% of the eyes allows patients to perform their daily living tasks and to live an independent life.
